# Screening tools for brief interventions in nonclinical settings

**DOI:** 10.1186/1940-0640-8-S1-A35

**Published:** 2013-09-04

**Authors:** Craig Jones, Carol Foster, Sarah Jones, Paul Jordan, John Bradley

**Affiliations:** 1 Public Health Wales, Cardiff, UK

## 

The NHS NICE Guidelines (2010) list a number of screening tools which have been validated for use with adults and young people (16+) for example AUDIT and AUDIT-C, CRAFFT, SASQ or FAST. The guidance states that the screening tool should be appropriate to the setting; for example, FAST would be appropriate in an emergency department. However, there is not a similar recommendation for Health and Social care settings. The aim of this project is to recommend a standardised method to be used in Wales for screening in nonclinical settings with Health and Social care partners. The Public Health Wales team have successfully undertaken a training programme so that everyone who has a 'teachable moment' has the skills and confidence to address hazardous or harmful drinking behaviours via an evidence based approach. They have been training across Wales in all Health and Social care settings. AUDIT-C is one of the screening tools recommended by NICE and the PHW team will undertake an evaluation of AUDIT-C as a 'method of choice' in nonclinical settings. The advantages and disadvantages of the AUDIT- C screening tool and a comparison with FAST will be undertaken. The initial stage would be a review of the literature and methodology available and an evaluation will be conducted. The review and recommendations will take place through 2013. PHW plan to ensure standardisation across Wales and to recommend a method of choice to nonclinicians.

